# Effect of size and location of simulated lytic lesions on the structural properties of human vertebral bodies, a micro-finite element study

**DOI:** 10.1016/j.bonr.2020.100257

**Published:** 2020-03-09

**Authors:** M.C. Costa, L.B. Bresani Campello, M. Ryan, J. Rochester, M. Viceconti, E. Dall'Ara

**Affiliations:** a Department of Oncology and Metabolism, Mellanby Centre for bone Research, University of Sheffield, UK; b INSIGNEO Institute for in silico Medicine, University of Sheffield, UK; c Academic Unit of Medical Education, Medical School, University of Sheffield, UK; d Department of Industrial Engineering, Alma Mater Studiorum - University of Bologna, Italy; e Medical Technology Lab, IRCCS Istituto Ortopedico Rizzoli, Bologna, Italy

**Keywords:** Spinal metastases, Lytic lesions, Biomechanics, Vertebral strength, Finite element, microFE, Parametric analysis, Mechanical properties

## Abstract

Currently, the Spinal Instability Neoplastic Score system is used in clinics to evaluate the risk of fracture in patients with spinal metastases. This method, however, does not always provide a clear guideline due to the complexity in accounting for the effect of metastatic lesions on vertebral stability. The aim of this study was to use a validated micro Finite Element (microFE) modelling approach to analyse the effect of the size and location of lytic metastases on the mechanical properties of human vertebral bodies. Micro Computed Tomography based microFE models were generated with and without lytic lesions simulated as holes within a human vertebral body. Single and multiple lytic lesions were simulated with four different sizes and in five different locations. Bone was assumed homogenous, isotropic and linear elastic, and each vertebra was loaded in axial compression. It was observed that the size of lytic lesions was linearly related with the reduction in structural properties of the vertebral body (reduction of stiffness between 3% and 30% for lesion volume between 4% and 35%). The location of lytic lesions did not show a clear effect on predicted structural properties. Single or multiple lesions with the same volume provided similar results. Locally, there was a homogeneous distribution of axial principal strains among the models with and without lytic lesions. This study highlights the potential of microFE models to study the effect of lesions on the mechanical properties of the human vertebral body.

## Highlights

• MicroFE models can show the effect of lytic lesions on vertebral properties.
• The size of the lesions was more critical than the location of the lesions.
• Lesions affecting the cortical shell had a larger effect on the local strains.
• Multiple lesions showed a similar effect to single lesions.

## Graphical abstract

**Figure:**
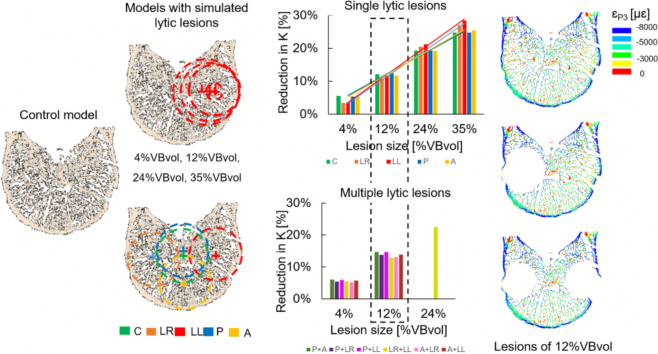

## 1 Introduction

Lytic lesions represent 95% of the spinal metastases developed at advanced stages of a cancer (Vialle et al., 2015). These lesions are described as focal regions of bone loss, which cause an increase in bone fragility and risk of pathological fractures (Burke et al., 2018; Hardisty et al., 2012; Ebihara et al., 2004). The clinical assessment of the risk of fracture of vertebrae with metastatic lytic lesions is based on the Spinal Instability Neoplastic Score (SINS) system, which, mainly qualitatively, evaluates six clinical and radiological data. Often, surgical consultation is required to support the SINS as in many cases it fails to provide a clear guideline (Vialle et al., 2015; Sutcliffe et al., 2013). The SINS system does not account for the effect of the structural properties of the lesion, as its size and location, over vertebral stability. Such parameters are already used to assess the instability of long bones affected by metastatic lesions (Mirel's scoring system), even though it remains to be investigated their relevance for the assessment of spinal instability.

Experimental studies show some controversy about the effect of the size of mechanically induced lytic lesions on the strength of human vertebrae. A few studies performed on human vertebrae tested under eccentric compression found no (Alkalay et al., 2018; Windhagen et al., 1997) or weak (R^2^ = 0.26, *n* = 45 from T3 to T12 Silva et al. (1993); and R^2^ = 0.51, *n* = 10 from T1 to L1 McGowan et al. (1993)) correlation between the size of induced lytic lesions and the vertebral failure. In particular, it was found that transcortical lesions caused a significant decrease in strength compared to induced lesions disrupting only the trabecular bone (McGowan et al., 1993; Silva et al., 1993). Recent experiments showed a significant increase in the values of principal strains distributed along the anterior surface of human vertebral bodies with induced lesions larger than 30% of the vertebral body volume. Moreover, a relationship between the progression of the strain pattern and the failure location was shown (Palanca et al., 2018).

Finite element (FE) models based on general geometries or clinical computed tomography (CT) images of human vertebrae, have been used to better understand the importance of the size and location of lytic lesions on the risk of vertebral burst fracture initiation (Galbusera et al., 2018; Tschirhart et al., 2004; Whyne et al., 2001, Whyne et al., 2003). In these computational studies it was shown that the size of lytic lesion is more critical than the location of the lesion for the risk of vertebral burst fractures (increase of 8-fold and 5% in axial displacements for lesions of up to 30% of the vertebral body volume and for lesions placed across the vertebral body, respectively) (Galbusera et al., 2018; Tschirhart et al., 2004). However, it is still unknown how lytic lesions affect the mechanical behaviour of the vertebral bone tissues, accounting for its heterogeneous microstructure. Furthermore, for some of the prognosis scoring systems mostly used in clinical practice to assess patients with spinal metastases, the number of lesions in the vertebral body is a critical parameter (Cassidy et al., 2018). For example, both Tokuhashi and Tomita prognosis scoring systems, recommend conservative treatments to patients with multiple lesions compared to those with single lesions (Parkes et al., 2018; Tokuhashi et al., 2005). Therefore, it is also important to study the effect of multiple lesions in the deterioration of spinal stability.

MicroCT based FE models (microFE) can be generated from high resolution images and, by resolving bone microstructure, these models can provide a more detailed understanding about the effect of diseases such as bone metastases on the local and structural properties of bones. These models have been recently used to evaluate the effect of structural changes due to osteoporosis before and after vertebroplasty (Badilatti et al., 2017). The local and structural predictions of microCT based FE models have been recently validated against time lapsed compressive test and digital volume correlation approaches for porcine vertebral bodies (Costa et al., 2017), human and bovine trabecular bone (Chen et al., 2017) and the mouse tibiae (Oliviero et al., 2018). Moreover, linear microFE based on high resolution peripheral CT (HR-pQCT) images have been found to accurately predict the structural properties of human vertebral body sections (R^2^ ≥ 0.88 for vertebral strength) (Dall'Ara, 2012; Pahr et al., 2011).

The aim of this study was to develop a computational framework to analyse the effect of the size and location of virtually simulated lytic lesions on the local and structural mechanical behaviour of human vertebrae.

## 2 Materials and methods

### 2.1 Sample preparation & scanning procedure

One cadaveric spine fixed in formaldehyde was acquired from a 78 years old male donor with no medical history of bone disease (Medical Teaching Unit of the University of Sheffield) under the approval of the ethics committee of the University of Sheffield (reference number 012716). The vertebral motion segments from T12 to L2 were isolated and the posterior elements were removed. The middle vertebra (i.e. L1) was scanned in saline solution with a microCT scanner (VivaCT 80, Scanco Medical, Bruttisellen, Switzerland) with the following parameters: voltage 70 kVp, intensity 114 mA, integration time 300 ms, and isotropic voxel size 39 μm, similar to (Hussein et al., 2012). The images were reconstructed using the software provided by the manufacturer and applying a beam hardening correction based on a phantom with 1200 mg HA/cc density. This protocol allowed the reconstruction of images similar to those used in a previous validation study (Costa et al., 2017).

### 2.2 Image processing

**Fig. 1:**
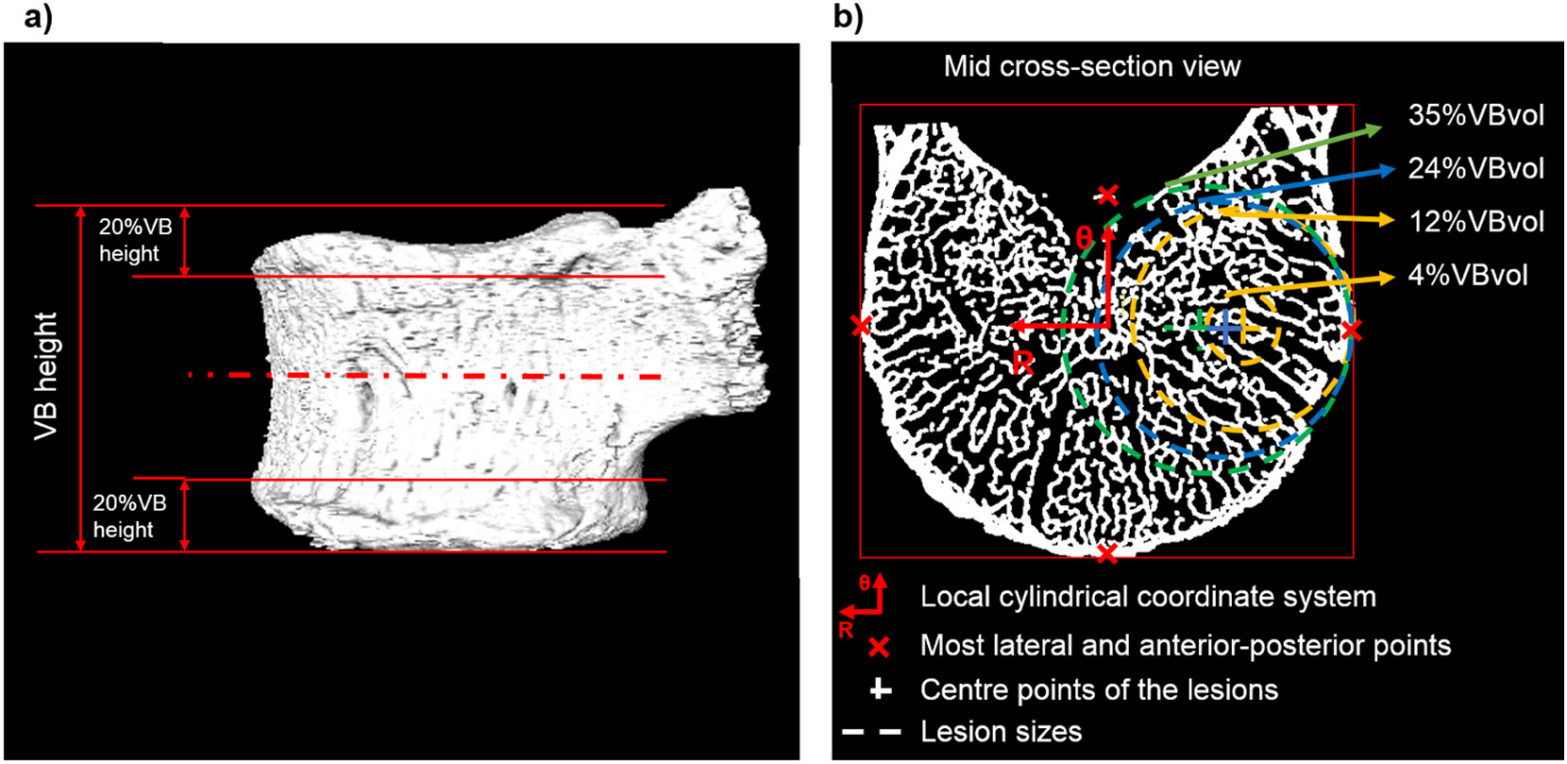
Left: Representation of the pre-processing operations performed for the definition of the vertebral body section model (cropping of the endplates in 20% of the vertebral body height). Right: Middle cross-section of the vertebral model, used to set up the position of the simulated lytic lesions in function of the distance between the geometric centre of the mid-section and the most lateral and anterior-posterior points (red crosses), and the size of the lesions. Illustration of lesions occupying 4%, 12% (orange), 24% (blue), and 35% (green) of the VBvol, placed over the lateral left compartment of the mid-cross section of the vertebral model.

From the reconstructed microCT image a section of the vertebral body was isolated, by removing 20% of its total height from each endplate (cranial and caudal) (Fig. 1). Before segmentation, a Gauss filter (kernel = 3 and σ = 1.2) (Chen et al., 2017) was applied. Segmentation was done using a single threshold value chosen by visual inspection of binary and grey-scale cross-section images as the value which best captured bone microarchitecture. A connectivity filter was used to remove bone voxels without face connectivity (Chen et al., 2017). The final binary image was then used to measure the bone volume (BV) of the specimen and a masked image of the vertebral body section was generated as described in Costa et al. (2017) to measure the total volume (VBvol) and the total bone volume fraction (Tot.BV/TV=BV/VBvol). From the masked image of the vertebral body, a surface object was generated and aligned along the transversal plane based on the in silico reference framework developed by Danesi et al. (2014). The rigid transformation was noted and applied to the model (Section 2.2) in order to avoid interpolation errors.

### 2.3 Finite element models

The “control model” was generated by converting every bone voxel of the binary image of the vertebral body section into an 8-noded linear hexahedral element. Bone was modelled as a homogeneous, isotropic, and linear elastic material with elastic tissue modulus equal to 12 GPa (Wolfram et al., 2010) and Poisson's ratio equal to 0.3. The model was aligned as described in Danesi et al. (2014), by applying the rigid transformation obtained from the image processing step. In particular, the cranio-caudal axis, was identified based on the concavity of the inferior vertebral notch. An axial compression of 1% apparent strain was applied to the nodes of the cranial section of the model, while the nodes of the caudal section were fixed in all directions.

From the control model 20 models were generated with simulated lytic lesions of different sizes and locations (Fig. 1). Simulated lytic lesions were modelled as spherical holes in the vertebral body volume. Lesions occupying 4%, 12%, 24%, and 35% of VBvol were simulated. Each lesion was placed in the central (C), lateral right (LR), lateral left (LL), anterior (A), or posterior (P) compartments of the mid cross-section of the vertebral body. The location of the centre of each lesion was defined with respect to a local cylindrical coordinate system set within the geometric centre of the mid cross-section of the model (Fig. 1b). The position of the most lateral, anterior and posterior points of the mid-section of the model was computed to define the radial distance (R) of the centre of each lesion from the origin of the reference system (Fig. 1b). The angular position (θ) of the centre of each lesion varied from 0° to 270° with increments of 90° (Fig. 1b). Lesions with VBvol equal to 12%, 24% and 35% had the centre in different positions, so that the lesions were tangent to the cortical shell of the vertebral body (Fig. 1). The centre of the lesion with VBvol equal to 4% was defined coincident to the centre of the lesions with VBvol equal to 12%. To test the effect of multiple lesions, models with two lesions of the same size were also created. In order to avoid overlap between lesions, only multiple lesions with a total VBvol equal to 4% (P + LR, P + LL, P + A, LR + LL, A + LR, A + LL), 12% (same as for VBvol equal to 4%) or 24% (LL + LR; the other configurations would include overlap between the lesions) were considered.

Each microFE model was simulated under the same boundary conditions of the control model. Spring stiffness (K) was computed for each model as the ratio between the sum of the axial reaction forces estimated from the caudal section of each model and the applied displacement. The ultimate force (F_U_) was estimated as the force required to deform 2% of the elements of the models (Pistoia et al., 2002) beyond the yield strain for compression (ɛ_p3Y_ = −8000 μɛ) or tension (ɛ_p1Y_ = 7200 μɛ) (Morgan et al., 2001). The percentage difference of the structural properties (K and F_U_) with and without simulated lytic lesions was estimated among the vertebral models. The distribution of the first and third principal strains was obtained from the middle 70% in height of the models with and without simulated lytic lesions. The control model was the largest model, it had 376 million degrees of freedom and took approximately 28 h to solve and post-process (Mechanical APDL v15.0, ANSYS, France) using parallel distributed memory with a maximum of 64 cores (High-Performance Computing cluster, Beagle, 2.70 GHz, 104 cores, 1.7 TB of RAM).

## 3 Results

**Fig. 2:**
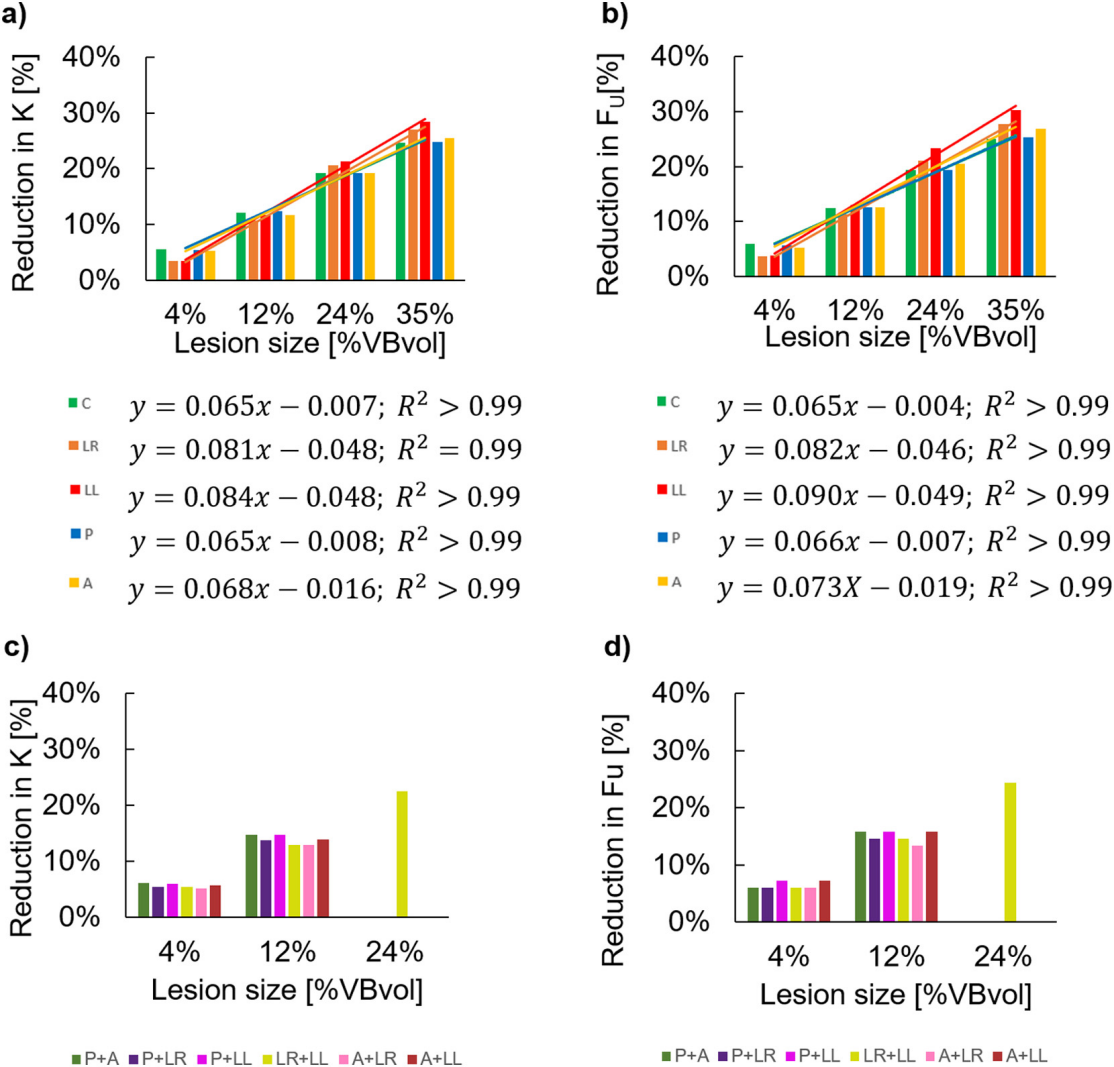
a)-b) Percentage reduction of predicted structural properties (spring stiffness, K, and ultimate force, F_U_) caused by single lytic lesions simulated with different sizes (4%, 12%, 24%, and 35% of the VBvol) and different locations (C: centre, LR: lateral right, LL: lateral left, P: posterior, and A: anterior). c)-d) Percentage reduction of predicted structural properties (spring stiffness, K, and ultimate force, F_U_) caused by multiple lytic lesions simulated with different sizes (4%, 12%, and 24% of the VBvol) and different locations (P + A: posterior and anterior, P + LR: posterior and lateral right, P + LL: posterior and lateral left, LR + LL: lateral right and left, A + LR: anterior and lateral right, A + LL: anterior and lateral left).

The interested readers can find the guideline about how to access the data related to this study here (https://doi.org/10.15131/ shef.data.11958954). The K and F_U_ for the control model and for those with simulated lesions are reported in Table 1. Strong linear relationships were found between the size of the simulated single lytic lesions and the decrease in predicted structural properties (R^2^ ≥ 0.99, intercept between −0.004 and −0.049%) (Fig. 2a–b). The decrease in structural properties (K and F_U_) caused by simulated single lytic lesions ranged from 3% to 30%. Single lesions occupying 4% and 12% of the VBvol showed similar reductions in structural properties regardless of their location (3%–13% reduction in K and Fu). Single lesions occupying 24% and 35% of the VBvol showed higher reduction in predicted structural properties (19%–30% reduction in K and F_U_) compared to smaller lesions occupying 4% or 12% of the VBvol (Table 1). For single lesions occupying different locations, only small differences, ranging between 2% and 5% in K and F_U_, were observed (Table 1).

**Table 1:** Structural properties (spring stiffness, K, and ultimate forces, F_U_) predicted from the vertebral models with and without simulated lytic lesions. Simulated lytic lesions grouped based on their sizes, and location (C: centre, LR: lateral right, LL: lateral left, P: posterior, and A: anterior).

Model ID	Lesion size [%VBvol]	Lesion(s) location	K [kN/mm]	%diff K [%]	Fu [kN]	%diff Fu [%]
Control	–	–	70.6	–	8.2	–
L#1	4%	C	66.6	6%	7.7	6%
L#2		LR	68.2	3%	7.9	4%
L#3		LL	68.1	4%	7.9	4%
L#4		P	66.8	5%	7.7	6%
L#5		A	66.9	5%	7.8	5%
L#42		P + LR	66.8	5%	7.7	6%
L#43		P + LL	66.4	6%	7.6	7%
L#45		P + A	66.3	6%	7.7	6%
L#23		LR + LL	66.7	6%	7.7	6%
L#52		A + LR	66.9	5%	7.7	6%
L#53		A + LL	66.6	6%	7.6	7%
L#6	12%	C	62.0	12%	7.2	12%
L#7		LR	63.0	11%	7.3	11%
L#8		LL	62.3	12%	7.1	13%
L#9		P	61.8	12%	7.2	13%
L#10		A	62.3	12%	7.2	13%
L#97		P + LR	60.9	14%	7.0	15%
L#98		P + LL	60.2	15%	6.9	16%
L#910		P + A	60.2	15%	6.9	16%
L#78		LR + LL	61.5	13%	7.0	15%
L#107		A + LR	61.4	13%	7.1	13%
L#108		A + LL	60.8	14%	6.9	16%
L#11	24%	C	57.0	19%	6.6	19%
L#12		LR	56.1	21%	6.5	21%
L#13		LL	55.6	21%	6.3	23%
L#14		P	57.0	19%	6.6	19%
L#15		A	57.0	19%	6.5	21%
L#1213		LR + LL	54.7	23%	6.2	24%
L#16	35%	C	53.1	25%	6.2	25%
L#17		LR	51.5	27%	5.9	28%
L#18		LL	50.5	28%	5.7	30%
L#19		P	53.1	25%	6.1	25%
L#20		A	52.6	25%	6.0	27%

Similar results were obtained between single and multiple lesions with the same total VBvol (Fig. 2c–d) equal to 4% (reduction in K in the range of 3–6% for single lesions and of 5–6% for multiple lesions; reduction in Fu in the range of 4–6% for single lesions and of 6–7% for multiple lesions), equal to 12% (reduction in K in the range of 11–12% for single lesions and of 13–15% for multiple lesions; reduction in Fu in the range of 11–13% for single lesions and of 13–16% for multiple lesions) or equal to 24% (reduction in K in the range of 19–21% for single lesions and of 23% for multiple lesions in LR + LL; reduction in Fu in the range of 19–23% for single lesions and of 24% for multiple lesions in LR + LL).

**Fig. 3:**
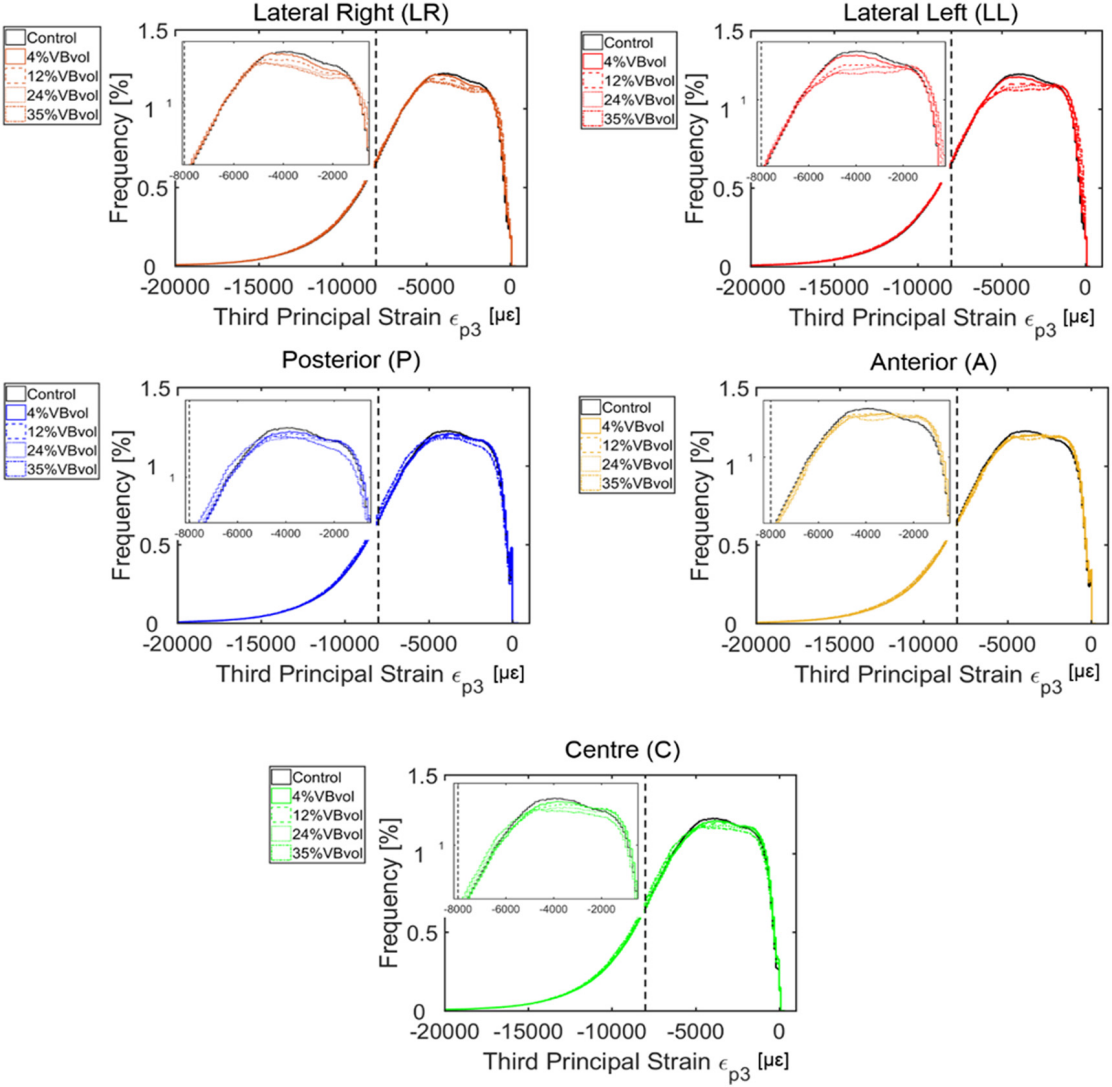
Distribution of the third principal strains obtained from the parametric models of simulated single lytic lesions based on the location (centre, C; lateral right, LR; lateral left, LL; anterior, A; and posterior, P) and size (4%, 12%, 24%, and 35% of the VBvol) of the lesions compared to the control model (solid black lines). Compressive yield strains, ε_P3Y_ = −8000 με (Morgan et al., 2001) are highlighted by vertical black dotted lines. Similar distributions were found for models with multiple lesions.

**Fig. 4:**
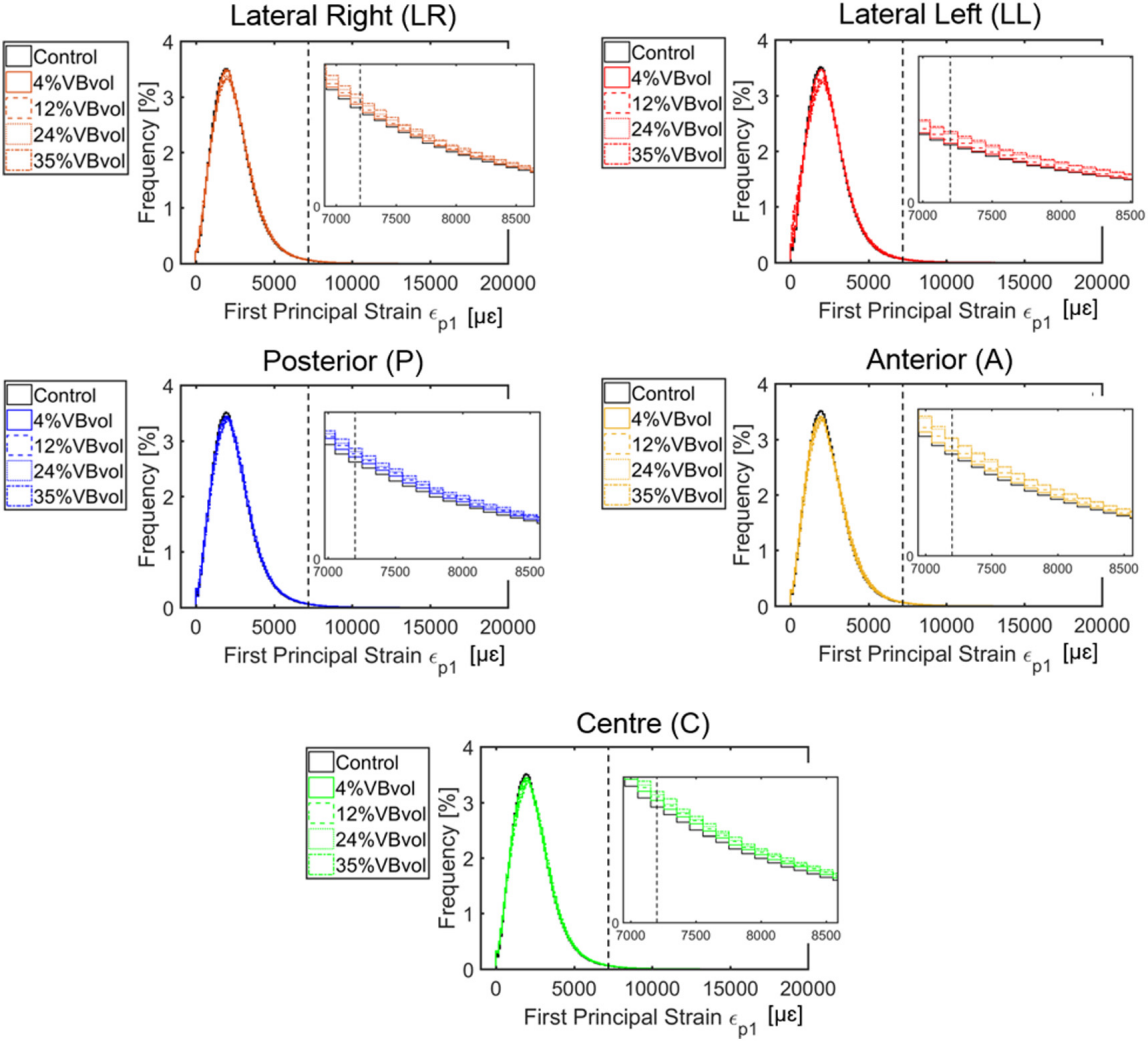
Distribution of the first principal strains obtained from the parametric models of simulated single lytic lesions based on the location (centre, C; lateral right, LR; lateral left, LL; anterior, A; and posterior, P) and size (4%, 12%, 24%, and 35% of the VBvol) of the lesions compared to the control model (solid black lines). Tensile yield strains, ε_P1Y_ = 7200 με (Morgan et al., 2001) are highlighted by vertical black dotted lines Similar distributions were found for models with multiple lesions.

At the local level, there were generally similar distributions of axial principal strains within the bone tissues of the models with and without simulated lytic lesions (mean ± standard deviation of −5000 ± 3000 μɛ for the third principal strains, and 2000 ± 1000 μɛ for the first principal strains) (Fig. 3 and Fig. 4). Similar distributions of strain were found for single or multiple lesions and regions of higher strain could be identified in models with larger lesions (Fig. 3 and Fig. 4).

**Fig. 5:**
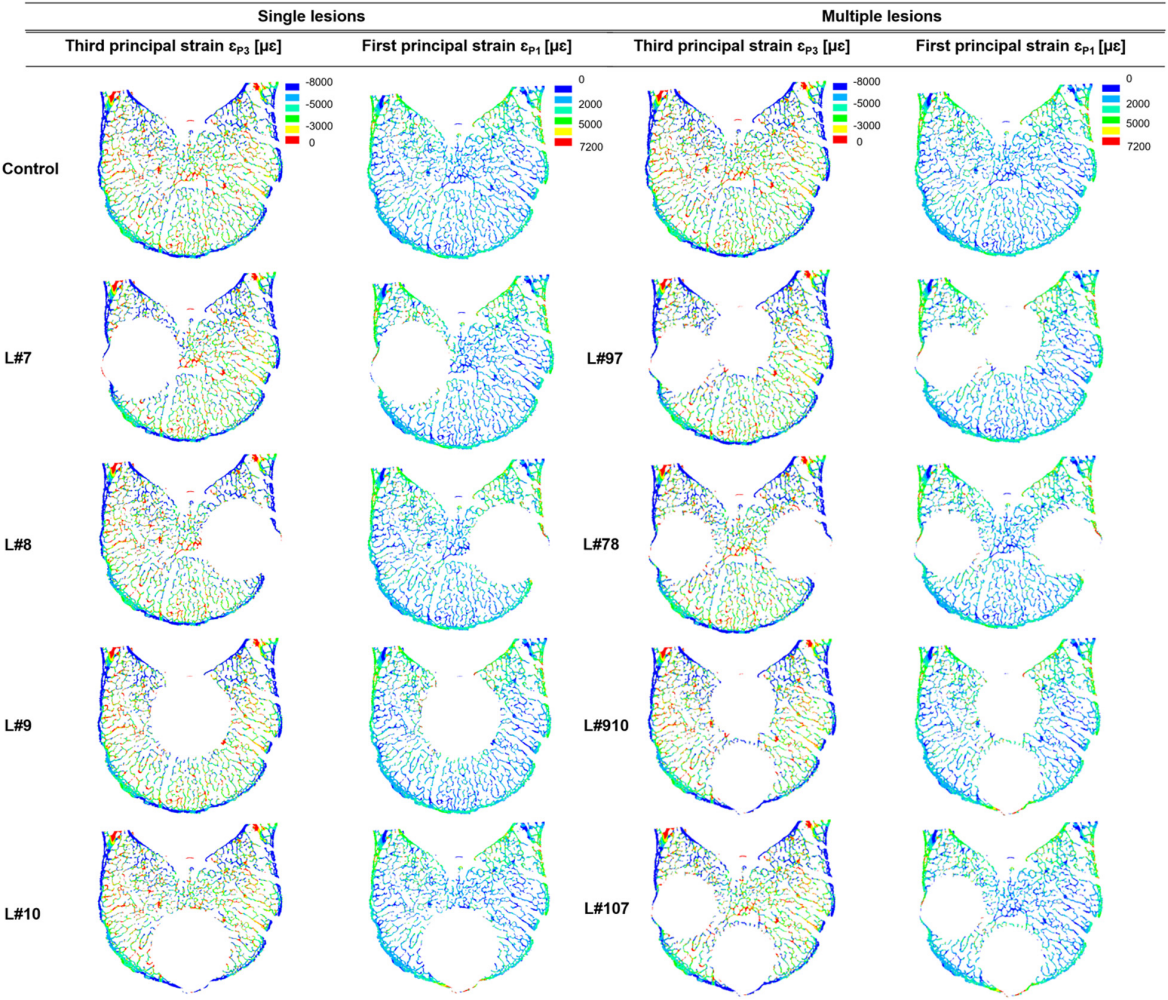
Distribution of third and first principal strain along the mid-cross section of the control model (top) against the models with single and multiple simulated lesions of 12% VBvol placed in the lateral right (L#7), lateral left (L#8), posterior (L#9), anterior (L#10), posterior and lateral right (L#97), lateral right and left (L#78), posterior and anterior (L#910), and anterior and lateral right (L#107) regions of the vertebral body.

**Fig. 6:**
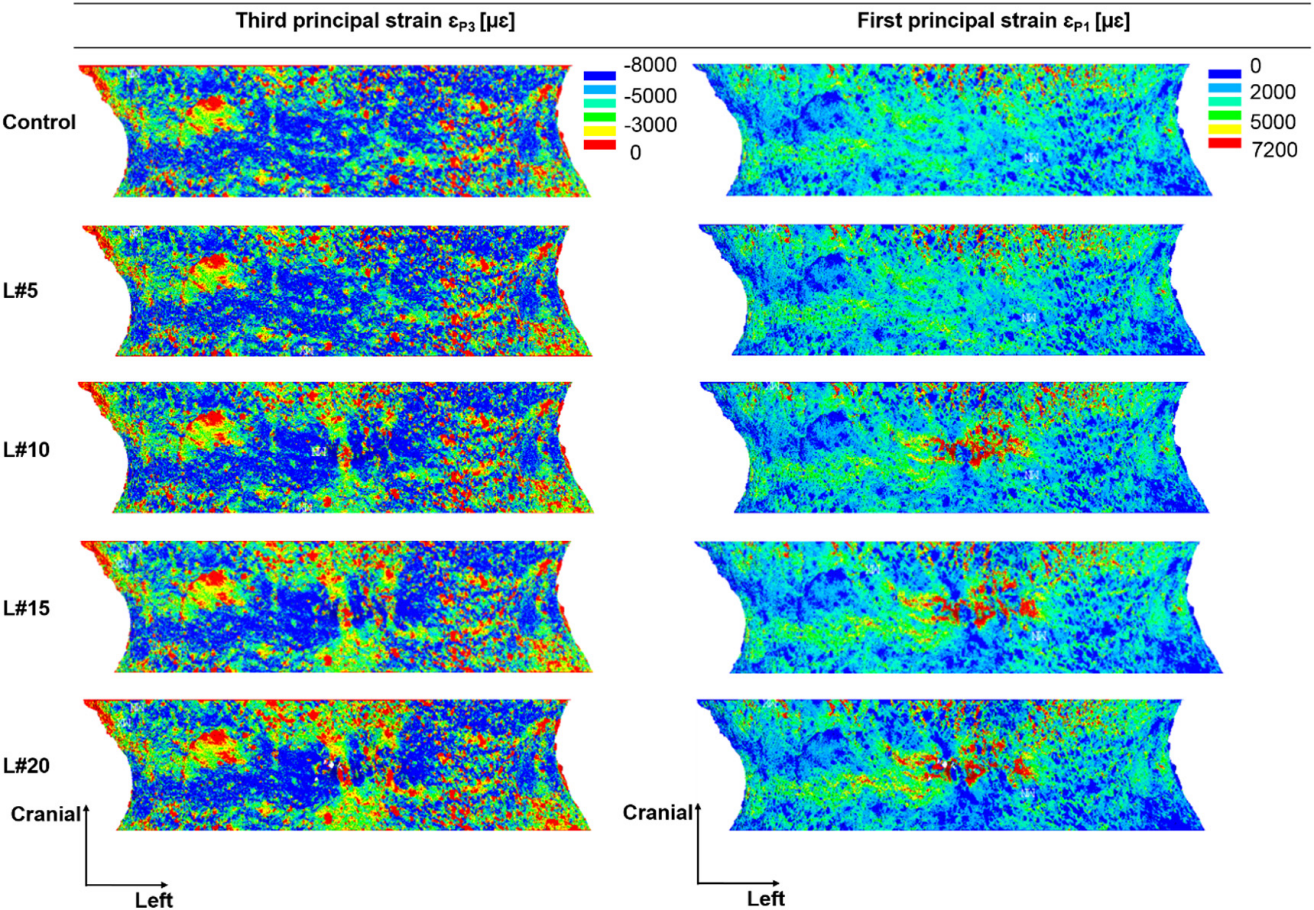
Distribution of third and first principal strains obtained from the middle 70% in height of the vertebral body. Plots show the frontal surface view of the control model (top) against the models with simulated lytic lesions of 4% VBvol (L#5), 12% VBvol (L#10), 24% VBvol (L#15), and 35% VBvol (L#20). Lesions located in the most anterior region of the vertebral body.

**Fig. 7:**
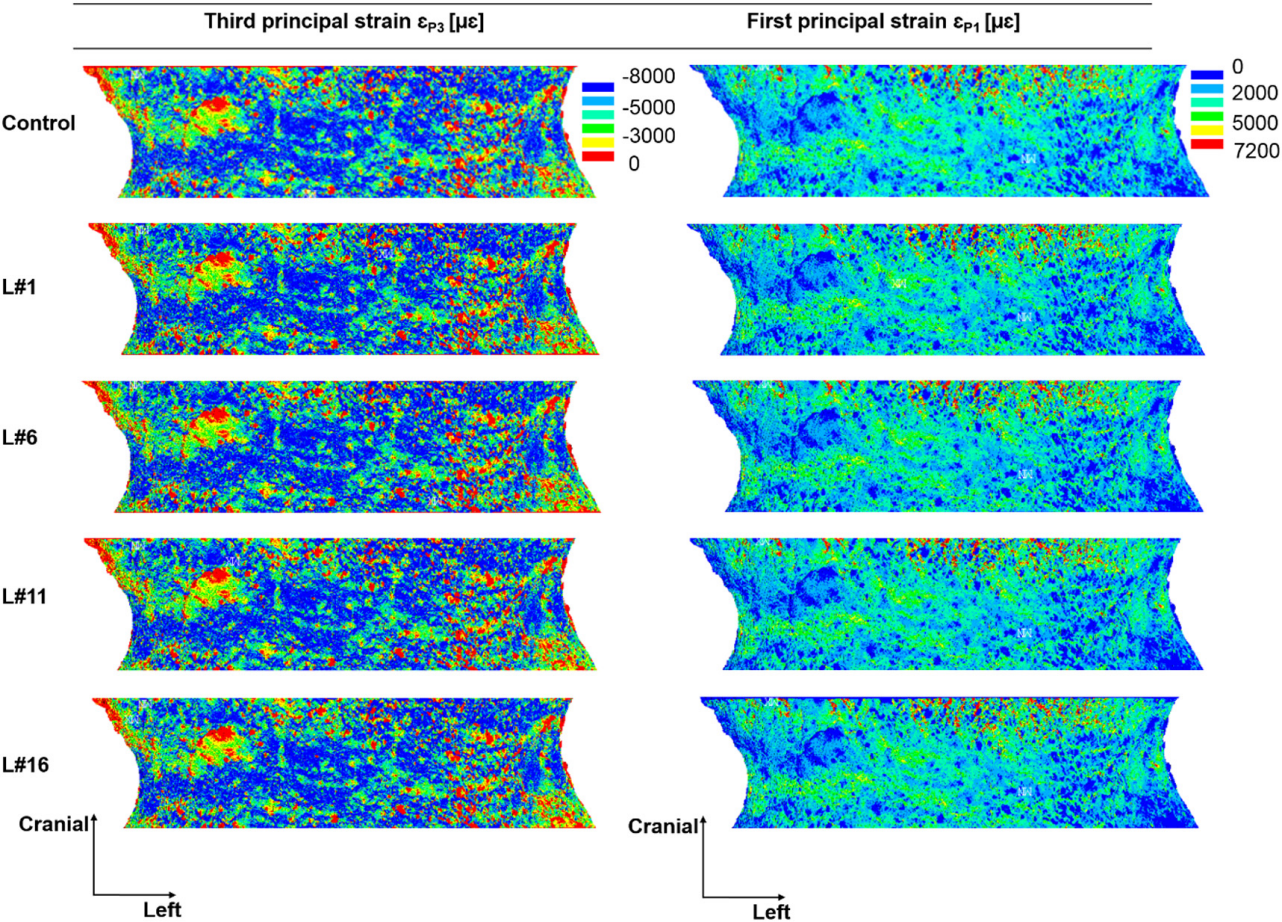
Distribution of third and first principal strains obtained from the middle 70% in height of the vertebral body. Plots show the frontal surface view of the control model (top) against the models with simulated lytic lesions of 4% VBvol (L#1), 12% VBvol (L#6), 24% VBvol (L#11), and 35% VBvol (L#16). Lesions located in the centre of the vertebral body.

Locally, high compressive strains were observed in the cortical shell of the vertebral models with and without simulated lytic lesions, for single and multiple lesions (Fig. 5). Lesions disrupting the cortical shell (i.e. larger or equal than 12% of the VBvol) located over the anterior (Fig. 6) and most lateral regions (Supplementary material Figs. S1.1 and S1.2) of the vertebral body showed a redistribution of the axial principal strains observed along the frontal surface of the vertebral body in comparison to the control model. Central and posterior lesions, did not cause any change in the strain distribution pattern observed along the frontal surface of the vertebral body compared to the control model (Fig. 7 and Supplementary material Fig. S1.3).

## 4 Discussion

This study aimed to develop a method to evaluate the effect of the size and location of simulated lytic lesions on the local and structural properties of the human vertebral body using a previously validated microFE modelling approach (Costa et al., 2017).

The results showed that the size of the simulated lytic lesions was linearly related to a decrease in predicted structural properties, with limited contribution of the position of the lesion or of multiple lesions scenario. Nevertheless, it remains to be investigated whether the same linear relationship would hold for larger simulated lytic lesions and for different specimens with different microarchitecture. These results are in line with previous studies, based on low resolution clinical images, which showed that the effect of the size of simulated lytic lesions on the vertebral mechanical properties is higher than that caused by the location of the lesions (Galbusera et al., 2018; Tschirhart et al., 2004; Whyne et al., 2001, Whyne et al., 2003; Windhagen et al., 1997; Silva et al., 1993).

At the local level, lytic lesions occupying up to 35% VBvol, had a minor impact on the distribution of axial principal strains, as these were similarly distributed among all the models with and without simulated lytic lesion (Fig. 3, Fig. 4). High principal compressive strains were mostly located in the cortical shell (Fig. 5). Contrary to the findings of Palanca et al. (2018) and Mizrahi et al. (1992) an increase in the mean or peak values of stresses and strains along the anterior surface of the models with lesions larger than 30% VBvol was not observed. The different findings from these studies can probably be explained by the different loading condition (compression vs eccentric compression towards the anterior side) and types of induced lytic lesions (internal vs drilled from the pedicles). Instead, in the present study it was observed a concentration of low principal strains in the surrounding bone tissues of the lesions, which was more pronounced for lesions larger or equal than 12% VBvol (Figs. 6 and S1.1 and S1.2). Such effect might result from the disruption of the cortical shell and consequently loss of connectivity in the tissue. These observations support the reduction in structural properties observed in the models with lytic lesions.

This study has a number of limitations. The simple compressive boundary conditions used in this study does not take into account for potential involvement and deformation of the endplates or of other relevant structures for the physiological loading distribution as the facet joints (Palanca et al., 2018; Ruspi et al., 2017). The lytic lesions were modelled as spherical holes and did not account for the heterogeneity in geometry and material properties of the material that fills the metastases or other lesions as bone cysts (Cox et al., 2011; Whyne et al., 2001; Whyne et al., 2000). Similar simplifications have been also used in experimental studies (Palanca et al., 2018; Alkalay and Harrigan, 2016; Alkalay, 2015; Silva et al., 1993) but may underestimate the effect of the lesions on the trabecular microstructure in their border. Furthermore, in this study we have assumed that the tissue around the lesion would not be affected by the lesion itself. Considering that higher density regions may be present in the vertebral bodies (Costa et al., 2019), it would be interesting to model the possible effect of densifications around the lesion. Nevertheless, in order to model accurately this phenomenon, bone remodelling (Badilatti et al., 2017) and lesion remodelling computational models should be used, that require a large amount of experimental data (apposition/resorption rates, position and geometrical properties of the densification, etc.) that at the moment is not available. Moreover, both improvements model the soft tissues within the lesion and the bone changes over time, will dramatically increase the number of elements and homogenised FE models (Pahr et al., 2011) would be probably a better approach to tackle this problem.

Nevertheless, the developed computational models have the advantage of considering for the first time the effect of lytic lesions on the trabecular microstructure alone or tangent to the external surface of the cortical shell, lesions that would be impossible to generate with standard experimental approaches. This study aimed to present the methodological approach and preliminary data on one specimen and, therefore, further analyses should be done to evaluate the effect of cortical involvement on the mechanical properties of the vertebral body. Nonetheless, its application to a large cohort of images acquired for different subjects has the potential of revealing the effect of different lesions' parameters on the mechanical properties of the metastatic vertebrae and to improve current scoring systems based on biomechanical assessment of vertebral structural stability.

In conclusion, the present study showed how a linear microFE modelling approach can be used to evaluate the effect of lytic lesions on the vertebral mechanical properties, and highlighted that the size of the lesion is the most important parameter to take into account when estimating the effect of lytic lesions on structural and local mechanical properties of the vertebral body. While, the generalization of these results should be supported by a larger dataset and more loading conditions in a future study, these results can be used to improve current scoring systems (e.g. SINS) by including in them a quantitative parameter as the size of the lesion, that could be measured from the medical images.

## Declaration of competing interest

The authors declare that they have no known competing financial interests or personal relationships that could have appeared to influence the work reported in this paper.

## Appendix A Supplementary data

Supplementary figures Image 1
