# Supplementary material for: Effect of size and location of simulated lytic lesions on the structural properties of human vertebral bodies, a micro-finite element study
Source: Bone Rep. 2020 Mar 9;12:100257. doi: 10.1016/j.bonr.2020.100257 (PMC7292861; doi:10.1016/j.bonr.2020.100257)
Supplement: Supplementary file 1 — Supplementary figures [file mmc1.docx]

**SUPPLEMENTARY MATERIAL**


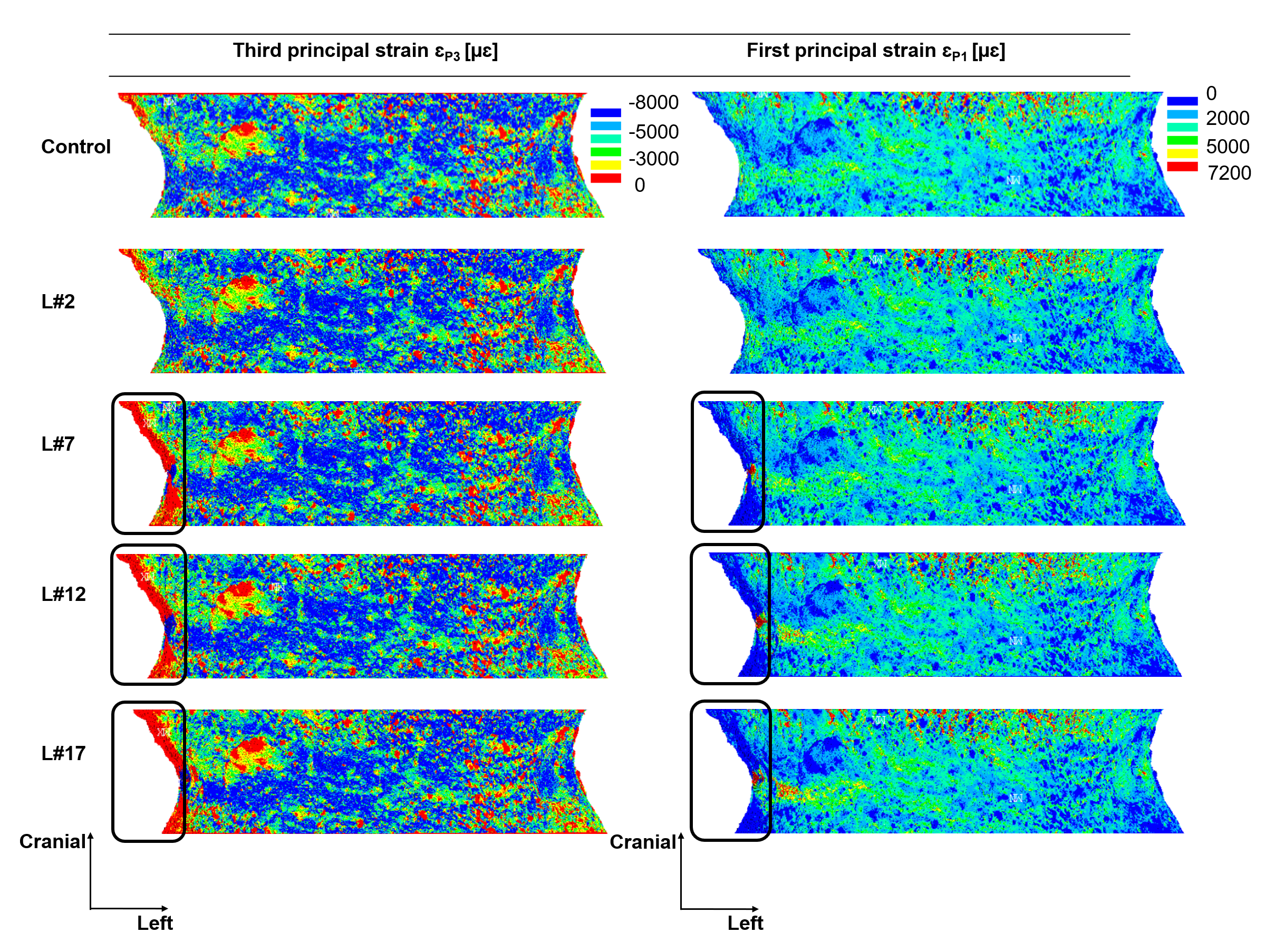


Fig. S1.1. Distribution of third and first principal strains obtained from the middle 70% in height of the vertebral body. Plots show the frontal surface view of the control model (top) against the models simulated lyic lesions of 4%VBvol (L#2), 12%VBvol (L#7), 24%VBvol (L#12), and 35%VBvol (L#17). Lesions located in the lateral right region of the vertebral body. Black arrows used to highlight differences to control model.


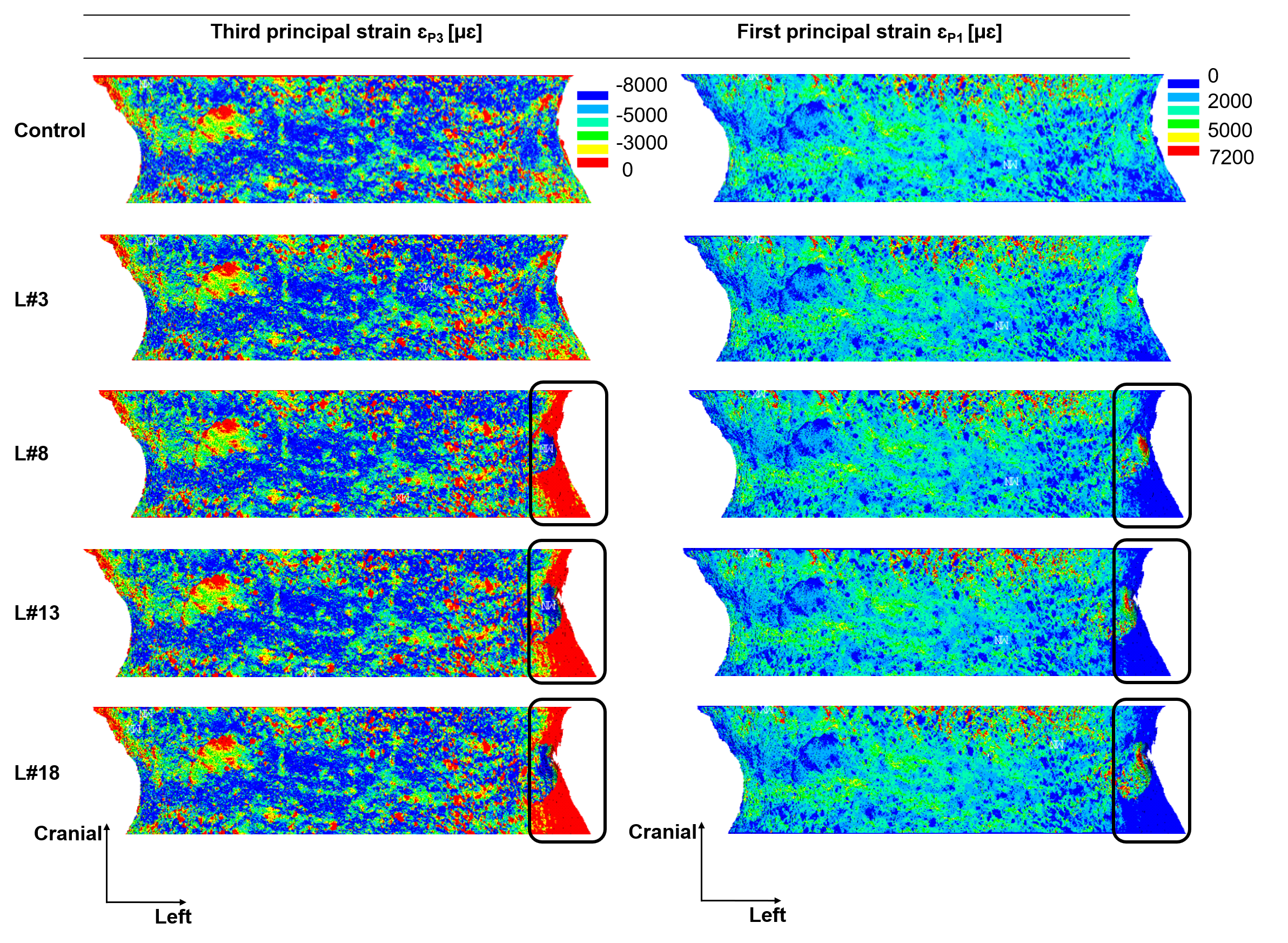


Fig. S1.2. Distribution of third and first principal strains obtained from the middle 70% in height of the vertebral body. Plots show the frontal surface view of the control model (top) against the models with simulated lyic lesions occupying 4% VBvol (L#3), 12% VBvol (L#8), 24% VBvol (L#13), and 35% VBvol (L#18). Lesions located in the lateral left region of the vertebral body. Black rectangle used to highlight differences to control model.


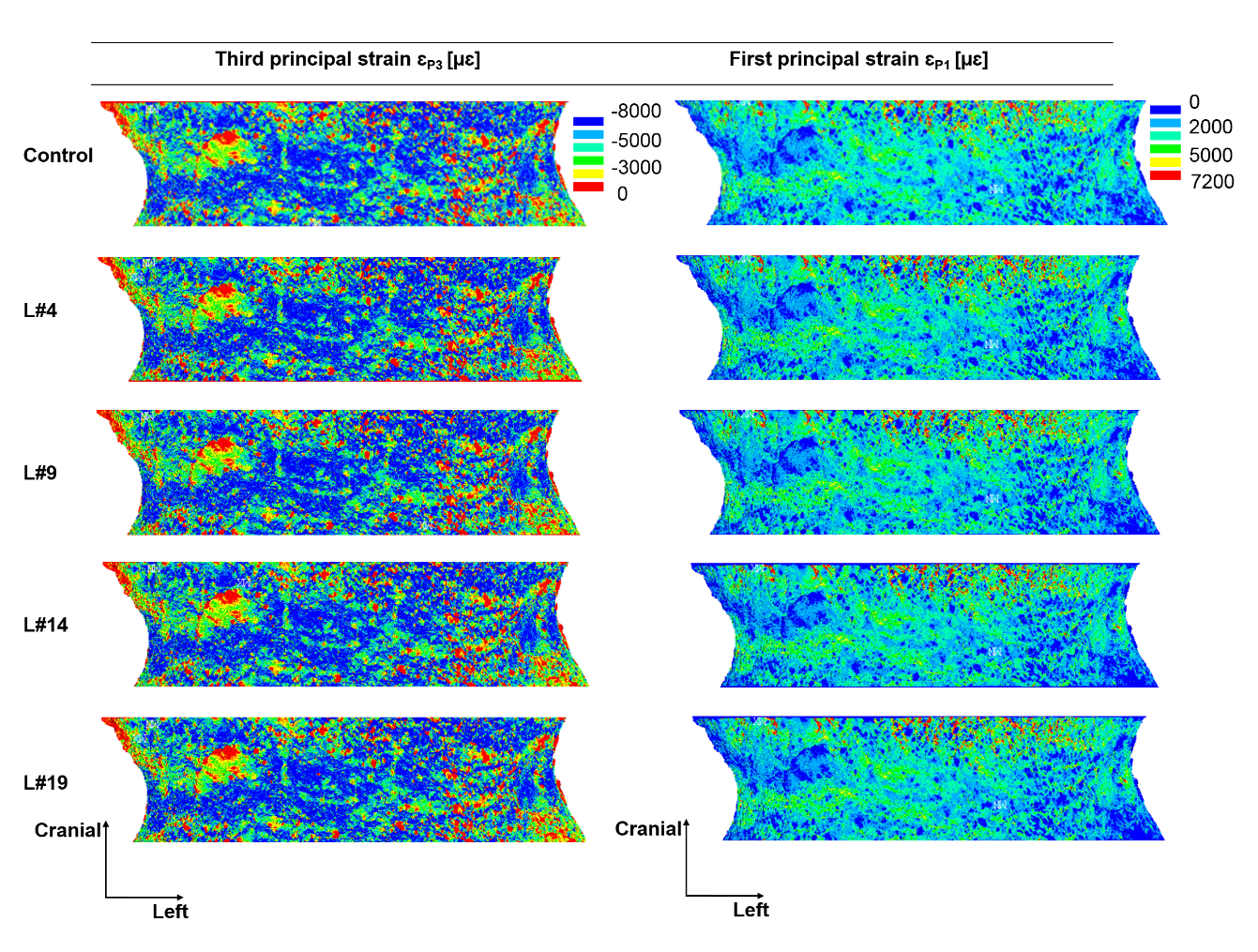


Fig. S1.3. Distribution of third and first principal strains obtained from the middle 70% in height of the vertebral body. Plots show the frontal surface view of the control model (top) against the models with simulated lyic lesions of 4%VBvol (L#4), 12%VBvol (L#9), 24%VBvol (L#14), and 35%VBvol (L#19). Lesions located in the most posterior region of the vertebral body.
